# COVID-19 vaccine acceptance among non-communicable disease patients in Haiti

**DOI:** 10.3389/fpubh.2025.1697746

**Published:** 2026-01-09

**Authors:** Evyrna Toussaint, Calixte Dawson, Darius L. Fénelon, Sarah M. Morris, Fediana Enoise, Rosemy Lolagne, Maurice Junior Chery, Kobel Dubique, Mary Clisbee, Davidson Laneau, Gene F. Kwan

**Affiliations:** 1 Zanmi Lasante, Croix-des-Bouquets, Haiti; 2 Department of Medicine, Boston University Chobanian and Avedisian School of Medicine, Boston, MA, United States; 3 Partners In Health, Boston, MA, United States; 4 Section of Cardiovascular Medicine, Boston Medical Center, Boston, MA, United States

**Keywords:** COVID-19, Haiti, hypertension, noncommunicable disease, vaccine acceptance, vaccine hesitancy

## Abstract

**Background/objectives:**

The COVID-19 pandemic continues to challenge global health, with individuals suffering from cardiometabolic conditions such as hypertension and diabetes at increased risk of severe complications. While vaccination is a key preventive measure, acceptance varies due to factors including safety concerns and cultural beliefs. This study assesses COVID-19 vaccine acceptance and hesitancy, along with knowledge, attitudes, and practices (KAP) related to COVID-19 among non-communicable disease (NCD) patients in rural central Haiti.

**Methods:**

We conducted a cross-sectional study from September 2021 to January 2022 at the outpatient NCD clinic of Hopital Universitaire de Mirebalais. A total of 229 NCD patients were surveyed to evaluate vaccine acceptance, hesitancy, and KAP regarding COVID-19. Data were analyzed using Chi-squared tests, T-tests, and logistic regression.

**Results:**

Participants’ average age was 56.2 years, with 75.1% being female. Vaccine acceptance was reported by 34% of unvaccinated individuals, with significant hesitancy among those favoring traditional medicine. Despite high awareness of COVID-19 (99.6%), accurate knowledge of symptoms and preventive measures was limited.

**Conclusion:**

Targeted interventions addressing vaccine hesitancy among people with non-communicable diseases in rural Haiti are crucial. Culturally sensitive education delivered through community health workers could improve vaccine uptake in this high-risk population.

## 1 Introduction

The global COVID-19 pandemic remains a significant burden worldwide. People living with cardiometabolic conditions such as hypertension, diabetes, and other non-communicable diseases (NCDs) are at higher risk of complications of COVID-19 (1). Non-communicable diseases (NCDs), particularly prevalent among the older adult population, increase the vulnerability to COVID-19 infection (2, 3). Furthermore, upon contracting the virus, those with chronic illnesses face significantly elevated risks of severe clinical outcomes or mortality compared to the general population (3). Even more remarkably, areas with higher NCD prevalence and socioeconomic disparities showed lower COVID-19 vaccination rates than regions with fewer NCDs and less socioeconomic inequality (4).

Vaccination is one of the principal interventions to prevent severe illness (5). The willingness of a population to adopt vaccines is a multifaceted issue influenced by various elements such as confidence in the safety and efficacy of vaccines, as well as personal, social, cultural, or religious beliefs and norms within that community (6, 7). Worldwide, 72% of individuals would be willing to receive the COVID-19 vaccine if it is considered safe and efficient, although willingness varies by nation (8). In a study carried out in three rural Haitian communities, the most prominent drivers of vaccine hesitancy were concerns about potential side effects and fear of contracting COVID-19 from the vaccine (9).

The most recent 2018 demographic health survey in Haiti revealed widespread hypertension and diabetes: among adults, 49% of women and 38% of men have high blood pressure, while 14% of women and 8% [3] of men have diabetes (10). Haiti initiated its COVID-19 vaccination campaign in July 2021, later than many other nations (9). However, by fall of 2022, only 2.2 percent of the Haitian populace had completed their COVID-19 vaccination, while fewer than 3.4 percent had received at least one dose of the vaccine (11).

While previous studies in Haiti have explored COVID-19 vaccine acceptance in the general population, there is limited information regarding this aspect among high-risk individuals, such as those living with NCDs. This study seeks to assess the degree of vaccine acceptance and barriers to vaccination among those living with NCDs in rural central Haiti, as well as identify relationships between vaccine acceptance and participant characteristics, and knowledge, attitudes, and practices (KAP) related to COVID-19.

## 2 Materials and methods

### 2.1 Study design

We conducted a cross-sectional study from September 2021 to January 2022 to assess COVID-19 vaccine acceptance and hesitancy, as well as the knowledge, attitudes, and practices (KAP) related to COVID-19 among individuals with non-communicable diseases (NCDs). The research was carried out at the outpatient NCD clinic of Hôpital Universitaire de Mirebalais (HUM). Established in 2013 through a partnership between the Haitian Ministry of Public Health and Zanmi Lasante / Partners In Health, HUM is located in the central plateau and serves a catchment area of approximately 3 million people. With a capacity of 300 beds, HUM provides both inpatient and outpatient care. The NCD clinic handles nearly 1,000 visits per month, focusing primarily on patients with hypertension, diabetes, heart failure, sickle cell disease, and chronic respiratory conditions.

### 2.2 Study population

The study sample was recruited during the first wave of the COVID-19 pandemic in Haiti. Participants were enrolled if they were followed in the NCD clinic at HUM and a scheduled appointment between September 2021 and January 2022. This included patients with hypertension, diabetes, heart failure, sickle cell disease, and chronic respiratory conditions, who were referred from primary care clinics for evaluation of a suspected NCD condition. Participants were aged 18 years or older and lived in the Central Plateau. Patients who were too ill to complete the survey were excluded.

Surveys were administered by a research team member and two trained NCD nurses. The nurses received detailed instructions on survey administration and data workflow. Each day, the nurses provided a list of scheduled patients, who were then individually approached for interviews before their clinical appointments. Participants were informed about confidentiality and provided consent before participating. Only those who agreed to participate completed the survey, and no compensation was offered.

### 2.3 Survey instrument

The research team developed a survey with closed-ended questions based on established instruments for assessing vaccine acceptance and hesitancy. These included prior surveys of COVID-19 knowledge and practices conducted in rural Haiti, including Mirebalais (9), and in rural Malawi among patients living with NCDs (12). The survey was initially created in English, translated into Haitian Creole, and pre-tested with people living in rural Haiti to ensure cultural and linguistic appropriateness. After finalizing the survey, it was integrated into the mobile electronic platform CommCare LTS 2.48.11 for data collection and management.

The survey comprised three main sections: (1) Demographic Characteristics, (2) Knowledge, Attitudes, and Practices (KAP) towards COVID-19, and (3) Vaccine Acceptance and Hesitancy. Demographic characteristics included age, gender, household status (e.g., head of household, spouse, child, parent, other), medical history (including conditions like hypertension, diabetes, heart disease, and asthma), marital status (e.g., married, single, widower, cohabitating, separated), education level (none, literacy, primary, secondary, higher), alcohol consumption, smoking status (current, former, never), employment type (e.g., agriculture, small business, other), and unemployment status due to COVID-19 (yes, no). To assess socioeconomic status, we employed multidimensional poverty measures developed by the Oxford Poverty and Human Development Initiative, which evaluates 10 indicators to measure the extent and severity of poverty (13), which we previously implemented in rural Haiti (10, 14). We report the proportion of indicators in which a participant is deprived. Participants were considered to have multidimensional poverty if they were deprived in 3 or more of the 10 indicators and experiencing severe poverty if deprived in 5 or more of the 10 indicators.

We assessed knowledge, attitudes, and practices (KAP) through 74 questions designed to gauge participants’ understanding and behavior regarding COVID-19. The Knowledge section featured questions on the transmission, spread, symptoms, and incubation period of COVID-19, with responses categorized as “yes,” “no,” or “I do not know.” The Attitudes section included two questions on beliefs about COVID-19 preventive measures, while the Practices section contained two questions evaluating adherence to behaviors that reduce virus transmission.

Additionally, the survey assessed vaccine acceptance, defined as the willingness to receive the COVID-19 vaccine if offered, and vaccine hesitancy, characterized by reluctance or refusal despite availability. Questions on current vaccination status complemented these measures.

Vaccine acceptance and hesitancy were assessed through two questions. We defined vaccine acceptance as having already been vaccinated or reporting willingness to take the vaccine should it be available. Participants who responded “No” or “I do not know” were classified as vaccine hesitant.

### 2.4 Data analysis approach

We conducted bivariate analyses to assess the relationship between vaccine acceptance and participant characteristics, COVID-19 knowledge level, beliefs about the pandemic, and self-reported safety guidelines adherence. Tests of significance included Chi-squared tests for categorical variables and independent T-tests for continuous variables. Variables found to be significant at the level of *α* = 0.05 in the bivariate analysis were selected for a multivariable logistic regression model. We used Cochran’s formula for finite populations to determine a target sample size of 222, allowing for a 5% margin of error within a 95% confidence interval, based on a population proportion of 50%. Analyses were conducted using Stata v17.0.

### 2.5 Ethics

This study was approved by the Institutional Review Boards of Zanmi Lasante (ZLIRB05212023, ZLIRB05202021) and Boston University Medical Campus (H-34326). We obtained written informed consent from all participants.

## 3 Results

### 3.1 Demographic and social characteristics

This study included 229 patients with NCDs. The average age of participants was 56.2 years (standard deviation 14.5), with 172 (75.1%) being female (Table 1; Supplementary Table S1). The most prevalent chronic conditions among participants were hypertension (159, 69.4%) and diabetes (114, 49.8%), with 57 participants (24.9%) having both conditions.

**Table 1:** Demographic characteristics.

	Vaccine acceptance		
Characteristic*	Yes	No/I do not know	*p*-value
N	89 (38.9%)	140 (61.1%)	
Age, mean years (sd)	57.6 (16.0)	55.3 (13.4)	0.252
Female sex	57 (64.0%)	115 (82.1%)	0.005
Formal education			0.427
None	22 (24.7%)	47 (33.6%)	
Literacy only	10 (11.2%)	11 (7.9%)	
Primary	29 (32.6%)	38 (27.1%)	
Secondary	23 (25.8%)	40 (28.6%)	
Higher	5 (5.6%)	4 (2.9%)	
Employment			0.667
Agriculture	15 (16.9%)	22 (15.7%)	
Small business	25 (28.1%)	48 (34.3%)	
Other	15 (16.9%)	17 (12.1%)	
Unemployed	34 (38.2%)	53 (37.9%)	
Tobacco use			0.001
Current/Former	23 (25.8%)	13 (9.4%)	
Never	66 (74.2%)	126 (90.7%)	
Alcohol use			0.239
Current/Former	18 (20.2%)	20 (14.3%)	
Never	71 (79.8%)	120 (85.7%)	
Chronic disease diagnoses			
Hypertension	67 (42.1%)	92 (57.9%)	0.126
Diabetes	41 (36.0%)	73 (64.0%)	0.370
Heart disease	8 (32.0%)	17 (68.0%)	0.456
Asthma	4 (30.1%)	9 (69.3%)	0.538
Other	4 (50.0%)	4 (50%)	0.511
None	0	3 (100%)	0.164
Multidimensional poverty			
Indicators deprived, mean (SD)	5.3 (1.44)	5.2 (1.56)	0.798
Multidimensional poverty†	85 (95.5%)	132 (94.3%)	0.686
Severe multidimensional poverty^#^ (*n*, %)	69 (77.5%)	101 (72.1%)	0.364

*, *n* (%), unless otherwise indicated; †, deprived in 3 of 10 indicators; #, deprived in 5 of 10 indicators.

Among the participants, 69 (30.1%) had no formal education and were not literate. Employment was distributed across small businesses (73, 31.9%) and agriculture (37, 16.2%), while 87 (38.0%) were unemployed. The prevalence of multidimensional poverty was 94.8% (217 participants), and 170 participants (74.2%) were experiencing severe poverty. Participants were deprived in a mean of 5.2 (SD 1.5) out of ten multidimensional poverty measures (Supplementary Table S2).

### 3.2 Vaccine acceptance

**Figure 1:**
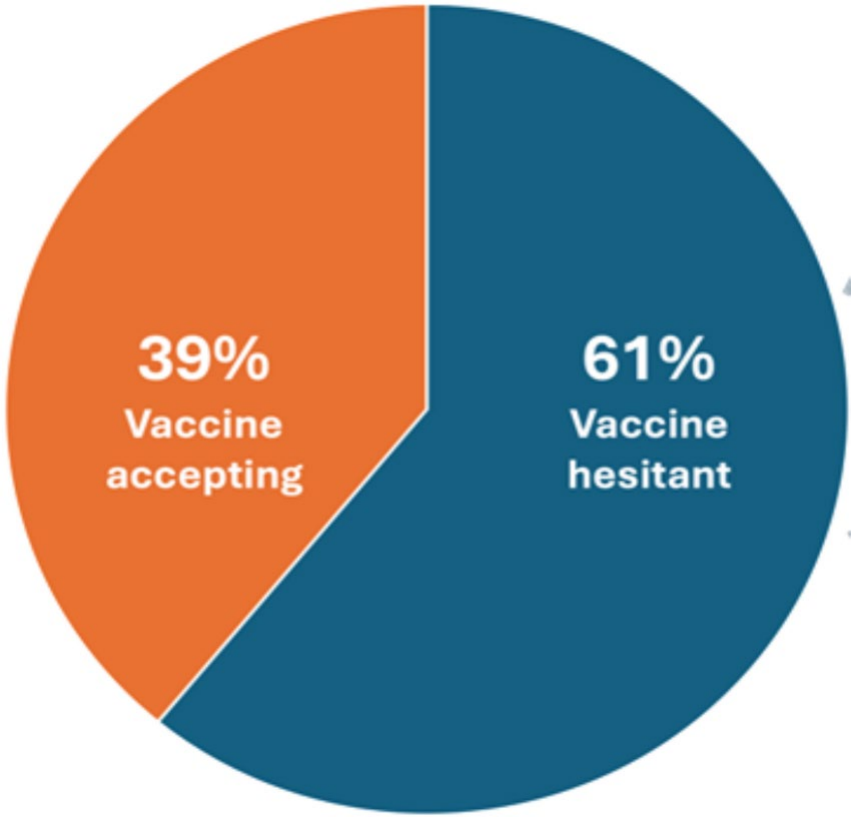
Vaccine acceptance and influencing factors among people with noncommunicable disease conditions in rural Haiti, *n* = 229.

Of 229 participants, we found that 89 (38.9%) were accepting of the COVID-19 vaccine as 14 were already vaccinated and 75 would take the vaccine if offered (Figure 1). 140 participants would not take the vaccine. No association was found between multidimensional poverty and vaccine acceptance. In univariate analysis, those who were less likely to be vaccine accepting were women (*n* = 57/89 [64.0%] vs. *n* = 115/140 [82.1%], *p* = 0.005), and never smokers (*n* = 66/89 [74.2%] vs. *n* = 126/140 [85.7%], *p* = 0.001). There was no difference in the proportions of participants who were accepting vs. not accepting based on age, education, employment, alcohol use, type of NCD, and poverty.

### 3.3 Knowledge of COVID-19

While 222 participants (99.6%) had heard of COVID-19 before the survey, only 35.8% accurately identified fever and cough as common symptoms (Table 2; Supplementary Table S3). Although 177 participants (77.3%) knew that COVID-19 could lead to lung disease or respiratory failure, only 59 (25.8%) recognized it could be fatal. Most participants understood that masks (55.9%) and avoiding crowded areas (61.6%) were effective preventive measures. A similar proportion knew that transmission occurs via physical contact or respiratory droplets from an infected person, including those who are asymptomatic. Identifying the duration between exposure and symptom development was answered with the least accuracy (44, 19.2%). The only significant difference between vaccine-accepting and hesitant participants was in the belief about transmission via contact (73.0% vs. 54.3%, *p* = 0.018). Knowledge that COVID-19 was transmitted via contact with others was higher among vaccine accepting participants (65, 73.0%) vs. hesitant (76, 54.3%, *p* = 0.018). All other responses were similar between vaccine accepting and hesitant participants.

**Table 2:** Knowledge regarding COVID-19.

	Vaccine acceptance		
Survey question	Yes (*n* = 89)	No/I do not know (*n* = 140)	*p*-value
Is fever a symptom of COVID-19?			0.376
Yes	54 (60.7%)	93 (66.4%)	
No	35 (39.3%)	47 (33.6%)	
Is cough a symptom of COVID-19?			0.551
Yes	43 (48.3%)	62 (44.3%)	
No	46 (51.7%)	78 (55.7%)	
Can someone catch COVID-19 through the saliva of others?			0.085
Yes	66 (74.2%)	85 (60.7%)	
No	6 (6.7%)	10 (7.1%)	
Do not know	17 (19.1%)	45 (32.1%)	
Can someone catch COVID-19 from physical contact with others?			0.018*
Yes	66 (74.2%)	85 (60.7%)	
No	6 (6.7%)	10 (7.1%)	
Do not know	17 (19.1%)	45 (32.1%)	
Can COVID-19 be fatal?			0.056
Yes	17 (19.1%)	42 (30.0%)	
No	60 (67.4%)	72 (51.4%)	
Do not know	12 (13.5%)	26 (18.6%)	
Can hand washing prevent infection?			
Yes	51 (57.3%)	76 (54.3%)	0.266
No	32 (36.0%)	45 (32.1%)	
Do not know	6 (6.7%)	19 (13.6%)	
Is self-isolation at home the best way to prevent spread?			0.884
Yes	40 (44.9%)	59 (42.1%)	
No	32 (36.0%)	51 (36.4%)	
Do not know	17 (19.1%)	30 (21.4%)	

### 3.4 Attitudes towards pandemic control

Over half of the participants (132, 58%) believed that most COVID-19 information circulating was false and that praying could prevent infection (125, 55%) (Supplementary Table S4). Less than half (94, 41%) feared infection for themselves or their family. Vaccine-accepting participants were more likely to believe that the Haitian government was managing the pandemic well (21% vs. 10%, *p* = 0.023), that the pandemic was under control in Haiti (23% vs. 7%, *p* = 0.001), and globally (29% vs. 14%, *p* = 0.007). Vaccine-accepting participants were also more likely to choose going to a hospital if infected (97% vs. 79%, *p* < 0.001). Over one-third of participants reported that they would drink tea to combat infection.

### 3.5 Practices regarding COVID-19 prevention and treatment

Vaccine-hesitant participants were less likely to follow social distancing guidelines strictly (21% vs. 37%, *p* = 0.049, Supplementary Table S5). Most participants (178, 77%) reported wearing a mask outside their home during the peak of the pandemic in Haiti before, with no significant association with vaccine hesitancy.

### 3.6 Vaccine uptake and hesitancy

Prior to the study, 14 (6%) participants had received a COVID-19 vaccine (Supplementary Table S6). Participant knowledge and attitudes to COVID-19 vaccines is shown in Table 3. Among the 215 who had not been vaccinated, 75 (34%) indicated they would accept it if offered, and 89 (41%) would recommend it to their family. Eighteen participants had not previously heard about the vaccine; 12 (67%) of them were open to learning more. Most participants learned about the vaccine via radio (150, 65%) or from friends and family (99, 43%), while 25 (11%) learned from community health workers.

**Table 3:** Vaccine knowledge and attitudes.

	Vaccine acceptance		
Survey question	Yes (*n* = 89)	No/I do not know (*n* = 140)	*p*-value
Who would you trust the most to help you decide whether to take the vaccine?			0.009*
Healthcare worker	89 (100%)	123 (87.9%)	
Family or friend	0	7 (5.0%)	
Community leader/priest	0	7 (5.0%)	
Government or MoH	0	3 (2.1%)	
Where did you learn about the vaccine?			
Radio	57 (64.0%)	93 (66.4%)	0.711
Friend/Family/Neighbor	37 (41.6%)	62 (44.3%)	0.686
Community health worker	13 (14.6%)	12 (8.6%)	0.153
Television	12 (13.5%)	10 (7.1%)	0.113
Loudspeaker	1 (1.1%)	1 (0.7%)	0.746
Reasons for not taking the vaccine			
Prefer to take local medicine	N/A	82 (59.0%)	N/A
COVID-19 infection is not as serious as people say	N/A	61 (43.6%)	N/A
Concerned about potential vaccine side effects	N/A	57 (40.7%)	N/A
Concerned the vaccine could kill me	N/A	52 (37.1%)	N/A
Concerned about getting COVID-19 from the vaccine	N/A	50 (35.7%)	N/A

Vaccine-accepting participants had low belief in the vaccine’s effectiveness, but still higher than those who were hesitant (36% vs. 9%, *p* < 0.001). Of the 75 unvaccinated but accepting participants, 87% preferred receiving the vaccine from a Haitian health worker rather than an international one. Among the 140 vaccine-hesitant participants, the most common reasons for hesitancy were a preference for local medicine (82, 59%), the belief that COVID-19 was not serious enough to warrant prevention (61, 44%), fear of potential side effects (57, 41%), and concerns that the vaccine could be fatal (52, 37%). Women were more likely to exhibit vaccine hesitancy compared to men (65.6% vs. 50.7%, *p* = 0.005).

On multivariable analysis, vaccine acceptance was associated with current/former smoking after adjustment for sex, age, education, alcohol use, and multidimensional poverty (Table 4). Participants with current/former smoking were more likely to be accepting of the vaccine (OR 2.93, *p* < 0.001).

**Table 4:** Multivariable analysis of factors associated with vaccine acceptance in rural Haiti.

Variable	Odds ratio (95% CI)	*p*-value
Sex		
Male	1	
Female	0.64 (0.32–1.26)	0.20
Age		
18–30	1	
31–45	1.96 (0.46–8.27)	0.46
46–60	0.71 (0.17–3.00)	0.17
60+	0.69 (0.16–2.95)	0.16
Education		
No formal education	1	
Literacy only	2.90 (1.00–8.47)	0.051
Primary education	1.47 (0.66–3.24)	0.34
Secondary education	0.81 (0.32–2.06)	0.66
Higher than secondary education	1.74 (0.36–8.43)	0.49
Employment		
Unemployed	1	
Agriculture	0.95 (0.39–2.31)	0.91
Small business	0.67 (0.31–1.45)	0.31
Other	1.95 (0.74–5.08)	0.18
Tobacco use		
Never	1	
Current/former	2.93 (2.37–15.63)	< 0.001
Alcohol use		
Never	1	
Current/former	0.67 (0.25–1.77)	0.42
Multidimensional poverty		
None	1	
Multidimensional poverty	1.03 (0.24–4.51)	0.97
Severe poverty	1.68 (0.41–6.88)	0.47

## 4 Discussion

This study explored vaccine acceptance for COVID-19, reasons for vaccine hesitancy, and the knowledge, attitudes, and practices (KAP) regarding the virus among people with non-communicable diseases (NCDs). At the time this survey was conducted in September 2021–January 2022, awareness of COVID-19 was nearly universal, though accurate knowledge of symptoms and preventive measures was limited. Only about one-third of participants would accept the vaccine if offered. Factors independently associated with vaccine acceptance in multivariable analysis include female sex, and never smoking. Most participants learned about COVID-19 through either the radio, friends and family, and community health workers.

In this study sample, 34% of participants indicated they would accept the vaccine if offered. This acceptance rate is higher than the 21% reported in the study by Maurice et al., also conducted in the same region of rural Haiti, but among a community-based sample (6). Possible explanations include the heightened awareness of risk of severe illness or death from COVID-19 infection among individuals with NCDs (15), which may make them more inclined to get vaccinated. Additionally, people with chronic diseases have more frequent interactions with the healthcare system. The participants in this study have regular visits every 1–3 months at the NCD clinic at Hôpital Universitaire de Mirebalais (HUM). Thus, they are more engaged than general community members, leading to greater trust in the healthcare system (16).

The vaccine acceptance rate among participants in our study is similar to a study in Ethiopia but is lower than acceptance rates reported in India and Bangladesh (17, 18) and other low- and middle-income countries (LMICs), where the average vaccine acceptance rate is around 80% (19, 20). This rate exceeds those in the USA (64.6%) and Russia (30.4%) (20). In our study, 34% of participants who had not yet taken the vaccine stated they would accept it if offered.

In LMICs, personal protection against COVID-19 is a major factor in vaccine acceptance. People living in rural Haiti often prioritize herbal remedies over conventional treatments (21). Similarly, we found over one-third of participants preferred herbal remedies to combat COVID-19. This reliance on traditional remedies may also negatively influence the vaccine acceptance rate in our study.

Furthermore, we identified several significant predictors of vaccine acceptance and hesitancy among NCD patients in our study, including gender, tobacco use, healthcare worker involvement in decision-making, low child mortality, and the number of rooms in the house. Female patients were more likely to express vaccine hesitancy, a finding that aligns with other studies on sex differences in COVID-19 vaccine acceptance (22). Participants who reported tobacco use were less likely to accept the COVID-19 vaccine, which is similar to other studies evaluating vaccination behavior among smokers (23).

Understanding individuals’ KAP is crucial for designing and implementing effective vaccination and health promotion programs. Poor COVID-19 knowledge is associated with negative attitudes and inadequate practices for virus prevention (24). While 99% of our participants had heard of COVID-19, their knowledge level was lower than in similar studies in LMICs. In part, concerns over vaccine safety may influence vaccine acceptance, as 65% of participants in a survey of Haitian community members feared contracting COVID-19 from the vaccine (9).

In LMICs, concerns about side effects account for 44% of vaccine hesitancy (20), while our study found that preference for traditional medicine was the most prevalent reason for reluctance. Overall, the KAP levels in our study were low. Although most participants had heard about COVID-19, fewer than 60% could correctly identify symptoms, incubation periods, and preventive measures. This is lower compared to studies in Africa and Asia (25, 26). Discrepancies can be attributed to differences in population characteristics; for instance, Saudi Arabian studies involved pregnant women accustomed to vaccination, and Ethiopian studies focused on healthcare workers who are younger and more engaged with COVID-19 prevention.

Our study offers valuable insights into the KAP levels among rural Haitian communities that can help health policy makers develop targeted strategies. To enhance vaccine uptake, public health strategies should focus on delivering vaccine education tailored to local beliefs and practices. Engaging community health workers in educational efforts and addressing specific safety concerns can help build trust and acceptance. Moreover, employing culturally sensitive approaches and providing clear, accessible information about the benefits of vaccination are crucial for overcoming resistance. Our findings may also be relevant for other countries with similar resources and sociodemographic characteristics as Haiti.

Our study has several limitations. First, this study was conducted at the end of 2021, a time when attitudes and behaviors toward COVID-19 were evolving since the early stages of the pandemic and may have continued to change. Therefore, comparing our findings with those from studies conducted during the pandemic’s early phase may not be entirely appropriate. Second, in our cross-sectional study, we were unable to measure changes in vaccine acceptance or hesitancy over time or establish causal relationships between significant factors and KAP levels. Third, the absence of open-ended questions limited the depth of the gathered information to fully understand the rationale for health-promoting behaviors. Fourth, we defined those who were not accepting of the vaccine to include those responding “I do not know” to being willing to take the vaccine if it were available. This may have introduced bias, as individuals who selected “No” could hold different perspectives from those who selected “I do not know.” Combining these two response categories may have led to response bias, potentially influenced by survey length or participant fatigue. Fifth, although the survey was adapted from established instruments previously used in rural Haiti and Malawi, it was not formally revalidated for this study population. As a result, the generalizability of our findings should be interpreted with caution. Sixth, we categorized that participants who had already received the vaccine as “accepting of vaccination,” assuming they would be willing to be vaccinated again. However, some previously vaccinated individuals may not wish to receive another dose. At the time of the study, vaccination in Haiti was mandated only for hospital workers and international travelers. None of our participants were hospital workers, and those in rural areas rarely traveled internationally. While some vaccinated participants may have been vaccinated for non-voluntary reasons (e.g., workplace or travel requirements), we retained them in the “acceptance” category to align with prior literature.

## 5 Conclusion

In this study of people with NCD conditions in rural Haiti, there was very high awareness of COVID-19 infection. However, knowledge, attitudes, and practices to promote health were modest. Only about one-third of participants reported that vaccination was acceptable particularly due to concerns about vaccine safety and a strong preference for traditional medicine. These barriers are further exacerbated by gaps in knowledge regarding the severity of COVID-19 and effective preventive measures. Addressing these factors can improve vaccination rates, better protect vulnerable populations from severe COVID-19 outcomes, and contribute to broader public health objectives in similar rural settings.

## Data Availability

The original contributions presented in the study are included in the article/Supplementary material, further inquiries can be directed to the corresponding author.

## References

[1] Nikoloski Z Alqunaibet AM Alfawaz RA Almudarra SS Herbst CH El-Saharty S . Covid-19 and non-communicable diseases: evidence from a systematic literature review. BMC Public Health. (2021) 21:1068. doi: 10.1186/s12889-021-11116-w, 34090396 PMC8178653

[2] Fauci AS Lane HC Redfield RR. Covid-19—navigating the uncharted. N Engl J Med. (2020) 382:1268–9. doi: 10.1056/NEJMe2002387, 32109011 PMC7121221

[3] Azarpazhooh MR Morovatdar N Avan A Phan TG Divani AA Yassi N . COVID-19 pandemic and burden of non-communicable diseases: an ecological study on data of 185 countries. J Stroke Cerebrovasc Dis. (2020) 29:105089. doi: 10.1016/j.jstrokecerebrovasdis.2020.105089, 32807484 PMC7315949

[4] Rizk JG Shaya FT. County-level COVID-19 vaccination rates, non-communicable diseases, and socioeconomic inequities: applying Syndemic theory to vaccines. J Health Care Poor Underserved. (2022) 33:1736–46. doi: 10.1353/hpu.2022.0134, 36341659

[5] Hussain S. Immunization and Vacinnation In: Huremović D., (ed.) Psychiatry of pandemics. Cham, Switzerland: Springer (2019) doi: 10.1007/978-3-030-15346-5_13

[6] MacDonald NE. Vaccine hesitancy: definition, scope and determinants. Vaccine. (2015) 33:4161–4. doi: 10.1016/j.vaccine.2015.04.036, 25896383

[7] Bertin P Nera K Delouvee S. Conspiracy beliefs, rejection of vaccination, and support for hydroxychloroquine: a conceptual replication-extension in the COVID-19 pandemic context. Front Psychol. (2020) 11:565128. doi: 10.3389/fpsyg.2020.565128, 33071892 PMC7536556

[8] Kateeb E Danadneh M Pokorna A Klugarová J Abdulqader H Klugar M . Predictors of willingness to receive COVID-19 vaccine: cross-sectional study of Palestinian dental students. Vaccines (Basel). (2021) 9:954. doi: 10.3390/vaccines9090954, 34579190 PMC8471090

[9] Chery MJ Dubique K Higgins JM Faure PA Phillips R Morris S . COVID-19 vaccine acceptance in three rural communes in Haiti: a cross-sectional study. Hum Vaccin Immunother. (2023) 19:2204048. doi: 10.1080/21645515.2023.2204048, 37157153 PMC10171132

[10] Kwan GF Yan LD Isaac BD Bhangdia K Jean-Baptiste W Belony D . High poverty and hardship financing among patients with noncommunicable diseases in rural Haiti. Glob Heart. (2020) 15:7. doi: 10.5334/gh.388, 32489780 PMC7218772

[11] USAID Haiti: health COVID-19 fact sheet. Washington, D.C.: United States Agency for International Development. (2022).

[12] Zaniku HR Aron MB Vrkljan K Tyagi K Ndambo MK Banda GM . COVID-19-related testing, knowledge and Behaviors among severe and chronic non-communicable disease patients in Neno District, Malawi: a prospective cohort study. Int J Environ Res Public Health. (2023) 20:5877. doi: 10.3390/ijerph20105877, 37239604 PMC10217925

[13] Sabina Alkire UKaNS. The Global Multidimensional Poverty Index (MPI): 2018 Revision. 2018. Available online at: https://ophi.org.uk/sites/default/files/OPHI_MPI_Meth_Note_46_vs3.pdf

[14] Yan LD Pierre-Louis D Isaac BD Jean-Baptiste W Vertilus S Fenelon D . Does distance from a clinic and poverty impact visit adherence for noncommunicable diseases? A retrospective cohort study using electronic medical records in rural Haiti. BMC Public Health. (2020) 20:1545. doi: 10.1186/s12889-020-09652-y, 33054756 PMC7556963

[15] Xu X Shi Z Zhou L Lin J Atlantis E Chen X . Impact of COVID-19 on risks and deaths of non-communicable diseases in the Western Pacific region. Lancet Reg Health West Pac. (2024) 43:100795. doi: 10.1016/j.lanwpc.2023.100795, 38456087 PMC10920048

[16] Hill KM Twiddy M Hewison J House AO. Measuring patient-perceived continuity of care for patients with long-term conditions in primary care. BMC Fam Pract. (2014) 15:191. doi: 10.1186/s12875-014-0191-8, 25477059 PMC4264317

[17] Kumari A Ranjan P Chopra S Kaur D Kaur T Kalanidhi KB . What Indians think of the COVID-19 vaccine: a qualitative study comprising focus group discussions and thematic analysis. Diabetes Metab Syndr. (2021) 15:679–82. doi: 10.1016/j.dsx.2021.03.021, 33813241 PMC7997146

[18] Abedin M Islam MA Rahman FN Reza HM Hossain MZ Hossain MA . Willingness to vaccinate against COVID-19 among Bangladeshi adults: understanding the strategies to optimize vaccination coverage. PLoS One. (2021) 16:e0250495. doi: 10.1371/journal.pone.0250495, 33905442 PMC8078802

[19] Wake AD. The acceptance rate toward COVID-19 vaccine in Africa: a systematic review and meta-analysis. Glob Pediatr Health. (2021) 8:2333794X211048738. doi: 10.1177/2333794X211048738, 34616860 PMC8488505

[20] Solis Arce JS Warren SS Meriggi NF Scacco A McMurry N Voors M . COVID-19 vaccine acceptance and hesitancy in low- and middle-income countries. Nat Med. (2021) 27:1385–94. doi: 10.1038/s41591-021-01454-y, 34272499 PMC8363502

[21] Thesnor V Cheremond Y Sylvestre M Meffre P Cebrian-Torrejon G Benfodda Z. Survey on the traditional use of medicinal herbs in Haiti: a study on knowledge, practices, and efficacy prevention. Plants (Basel). (2024) 13:2383. doi: 10.3390/plants13172383, 39273867 PMC11396795

[22] Sileo KM Hirani IM Luttinen RL Hayward M Fleming PJ. A scoping review on gender/sex differences in COVID-19 vaccine intentions and uptake in the United States. Am J Health Promot. (2024) 38:242–74. doi: 10.1177/08901171231200778, 37847250 PMC10802093

[23] Krebs NM D'Souza G Bordner C Allen SI Hobkirk AL Foulds J . COVID-19 vaccination uptake and hesitancy among current tobacco users. Tob Use Insights. (2021) 14:1179173X211068027. doi: 10.1177/1179173X211068027 PMC8721404 34987300

[24] Bekele F Sheleme T Fekadu G Bekele K. Patterns and associated factors of COVID-19 knowledge, attitude, and practice among general population and health care workers: a systematic review. SAGE Open Med. (2020) 8:2050312120970721. doi: 10.1177/2050312120970721, 33240497 PMC7675903

[25] Yisak H Ambaw B Belay E Desalegn T Getie A Asrat M . Knowledge, attitude, acceptance, and practice of COVID-19 vaccination and associated factors complemented with constructs of health belief model among the general public in South Gondar, Ethiopia: a community-based study. Front Public Health. (2022) 10:914121. doi: 10.3389/fpubh.2022.914121, 36466498 PMC9714612

[26] Ashour HA Alhinti SF Hawsaoi SA Alsuwailem AA AlFarhan A Abdulmajeed I. Knowledge, attitude, and practice (KAP) of COVID-19 vaccine among Saudi mothers. Cureus. (2023) 15:e36826. doi: 10.7759/cureus.36826, 37123733 PMC10139822

