# Supplementary material for: COVID-19 vaccine acceptance among non-communicable disease patients in Haiti
Source: Front Public Health. 2026 Jan 9;13:1697746. doi: 10.3389/fpubh.2025.1697746 (PMC12827755; doi:10.3389/fpubh.2025.1697746)
Supplement: Supplementary file 1 [file Supplementary_file_1.docx]

Supplementary Materials

**Title: COVID-19 Vaccine Acceptance Among Non-Communicable Disease Patients in Haiti**

**Contents**

Supplementary Table S1. Demographic characteristics

Supplementary Table S2. Multidimensional Poverty Index

Supplementary Table S3. Knowledge regarding COVID-19

Supplementary Table S4. Beliefs regarding COVID-19

Supplementary Table S5. Practices relating to COVID-19

Supplementary Table S6. Vaccine attitudes

Supplementary Table S7. Fear of COVID infection

| **Supplementary Table S1. Demographic characteristics** | | | | |
| --- | --- | --- | --- | --- |
|  | **Vaccine Acceptance** | |  |  |
| **Characteristic** | **Yes (n = 89)** | **No/ I don’t know (n = 140)** | **P-value** |  |
| Interviewee |  |  | 0.88 |  |
| Head of household | 72 (80.9%) | 115 (82.1%) |  |  |
| Spouse of head of house | 6 (6.7%) | 10 (7.1%) |  |  |
| Child of head of household | 4 (4.5%) | 4 (2.9%) |  |  |
| Parent of head of household | 3 (3.4%) | 7 (5.0%) |  |  |
| Other | 4 (4.5%) | 4 (2.9%) |  |  |
| Marital status |  |  | 0.66 |  |
| Single | 17 (19.1%) | 32 (22.9%) |  |  |
| Widower | 18 (20.2%) | 20 (14.3%) |  |  |
| Cohabitating | 14 (15.7) | 17 (12.1%) |  |  |
| Separated | 5 (5.6%) | 9 (6.4%) |  |  |

| **Supplementary Table S2. Multidimensional Poverty Index** | | | |
| --- | --- | --- | --- |
|  | **Vaccine Acceptance** | |  |
| **Poverty category, n (%) deprived** | **Yes (n = 89)** | **No/ I don’t know (n = 140)** | **P-value** |
| Nutrition | 53 (59.6%) | 70 (50.0%) | 0.16 |
| Child Mortality | 0 | 7 (5.0%) | 0.032 |
| Years of school | 22 (24.7%) | 33 (23.6%) | 0.84 |
| School attendance | 10 (11.2%) | 18 (12.9%) | 0.72 |
| Cook fuel | 81 (91.0%) | 126 (90.0%) | 0.80 |
| Sanitation | 64 (71.9%) | 89 (63.6%) | 0.19 |
| Water | 65 (73.0%) | 111 (79.3%) | 0.27 |
| Electricity | 66 (74.2%) | 96 (68.6%) | 0.37 |
| Housing | 43 (48.3%) | 82 (58.6%) | 0.13 |
| Assets | 66 (74.2%) | 100 (71.4%) | 0.65 |

| **Supplementary Table S3. Knowledge regarding COVID-19** | | | |
| --- | --- | --- | --- |
|  | **Vaccine Acceptance** | |  |
| **Survey question** | **Yes (n = 89)** | **No/ I don’t know (n = 140)** | **P-value** |
| Have you heard of COVID-19? | 89 (100%) | 139 (99.3%) | 0.42 |
| Do you know the symptoms of COVID-19? | 71 (79.8%) | 109 (77.9%) | 0.71 |
| Are the following symptoms of COVID-19? | | |  |
| Fever | 54 (60.7%) | 93 (66.4%) | 0.38 |
| Cough | 43 (48.3%) | 62 (44.3%) | 0.55 |
| Flu | 26 (29.2%) | 38 (27.1%) | 0.73 |
| Loss of taste | 10 (11.2%) | 10 (7.1%) | 0.29 |
| Diarrhea | 10 (11.2%) | 11 (7.9%) | 0.34 |
| Sore throat | 5 (5.6%) | 9 (6.4%) | 0.80 |
| Can COVID-19 cause lung disease and respiratory failure? | | | 0.066 |
| Yes | 76 (85.4%) | 101 (72.1%) |  |
| No | 1 (1.1%) | 3 (2.1%) |  |
| Don’t know | 12 (13.5%) | 36 (25.7%) |  |
| Can fabric or surgical masks prevent COVID-19 infection? | 54 (60.7%) | 74 (52.9%) | 0.25 |
| Can BCG vaccination prevent infection? | | | 0.20 |
| Yes | 12 (13.5%) | 10 (7.1%) |  |
| No | 30 (33.7%) | 43 (30.7%) |  |
| Don’t know | 47 (52.8%) | 87 (62.1%) |  |
| Do you think there is no cure for COVID-19? | | | 0.33 |
| Yes | 37 (41.6%) | 45 (32.1%) |  |
| No | 11 (12.4%) | 18 (12.9%) |  |
| Don’t know | 41 (46.1%) | 77 (55.0%) |  |
| Can you get COVID-19 from eating or handling wild animals? | | | 0.076 |
| Yes | 19 (21.4%) | 27 (19.3%) |  |
| No | 24 (27.0%) | 22 (15.7%) |  |
| Don’t know | 46 (51.7%) | 91 (65.0%) |  |
| Can you get COVID-19 from someone with no fever? | | | 0.066 |
| Yes | 71 (79.8%) | 95 (67.9%) |  |
| No | 7 (7.9%) | 10 (7.1%) |  |
| Don’t know | 11 (12.4%) | 35 (25.0%) |  |
| Is it important to prevent children and young people from getting COVID-19? | | | 0.17 |
| Yes | 87 (97.8%) | 129 (92.1%) |  |
| No | 0 | 3 (2.1%) |  |
| Don’t know | 2 (2.3%) | 8 (5.7%) |  |
| Is it important to avoid going to crowded places to prevent infection? | | | 0.27 |
| Yes | 55 (61.8%) | 86 (61.4%) |  |
| No | 28 (31.5%) | 36 (25.7%) |  |
| Don’t know | 6 (6.7%) | 18 (12.9%) |  |

| **Supplementary Table S4. Beliefs regarding COVID-19** | | | |
| --- | --- | --- | --- |
|  | **Vaccine Acceptance** | |  |
| **Survey question** | **Yes (n = 89)** | **No/ I don’t know (n = 140)** | **P-value** |
| How much of the news and information about COVID-19 is made up? | | | 0.45 |
| A lot | 56 (62.9%) | 78 (55.7%) |  |
| Some | 17 (19.1%) | 22 (15.7%) |  |
| Not much | 9 (10.1%) | 21 (15.0%) |  |
| Not at all | 0 | 1 (0.7%) |  |
| Don’t know | 7 (7.9%) | 18 (12.9%) |  |
| Do you agree praying prevents infection? | | | 0.080 |
| Agree | 49 (55.1%) | 76 (54.3%) |  |
| Disagree | 37 (41.6%) | 48 (34.3%) |  |
| Don’t know | 3 (3.4%) | 16 (11.4%) |  |
| Describe your level of agreement: The Haitian government is handling the pandemic well | | | 0.023 |
| Strongly agree | 0 | 2 (1.4%) |  |
| Agree | 19 (21.4%) | 12 (8.6%) |  |
| Neutral | 20 (22.5%) | 24 (17.1%) |  |
| Disagree | 30 (33.7%) | 67 (47.9%) |  |
| Strongly disagree | 20 (22.5%) | 35 (25.0%) |  |
| Describe your level of agreement: I am afraid my family and I will be infected | | | 0.11 |
| Strongly agree | 10 (11.2%) | 10 (7.1%) |  |
| Agree | 35 (39.3%) | 39 (27.9%) |  |
| Neutral | 0 | 4 (2.9%) |  |
| Disagree | 28 (31.5%) | 51 (36.4%) |  |
| Strongly disagree | 16 (18.0%) | 36 (25.7%) |  |
| Describe your level of agreement: The pandemic is well-controlled in Haiti | | | 0.001 |
| Yes | 21 (23.6%) | 10 (7.1%) |  |
| No | 37 (41.6%) | 56 (40.0%) |  |
| Don’t know | 31 (34.8%) | 74 (52.9%) |  |
| Describe your level of agreement: The pandemic has been well-controlled globally | | | 0.007 |
| Yes | 26 (29.2%) | 20 (14.3%) |  |
| No | 30 (33.7%) | 43 (30.7%) |  |
| Don’t know | 33 (37.1%) | 77 (55.0%) |  |
| What actions would you take if you were infected with COVID-19? Participants selected more than one | | |  |
| Go to hospital | 86 (96.6%) | 111 (79.3%) | <0.001 |
| Drink herbal tea | 26 (29.2%) | 50 (35.7%) | 0.31 |
| Visit a priest | 3 (3.4%) | 6 (4.3%) | 0.73 |
| Stay home | 4 (4.5%) | 5 (3.6%) | 0.73 |

| **Supplementary Table S5. Practices relating to COVID-19** | | | |
| --- | --- | --- | --- |
|  | **Vaccine Acceptance** | |  |
| **Survey question** | **Yes (n = 89)** | **No/ I don’t know (n = 140)** | **P-value** |
| To what extent did you follow social distancing guidelines proposed by the government? | | | 0.049 |
| To a great extent | 33 (37.1%) | 29 (20.7%) |  |
| To a moderate extent | 3 (3.4%) | 9 (6.4%) |  |
| To some extent | 44 (49.4%) | 82 (58.6%) |  |
| Not at all | 6 (6.7%) | 8 (5.7%) |  |
| Declined to answer | 3 (3.4%) | 12 (8.6%) |  |
| Did you wear a mask when you left your home when the pandemic was at its worst | | | 0.280 |
| Yes | 70 (78.7%) | 108 (77.1%) |  |
| No | 15 (16.9%) | 30 (21.4%) |  |
| Did not leave home | 4 (4.5%) | 2 (1.4%) |  |

| **Supplementary Table S6. Vaccine attitudes** | | | |
| --- | --- | --- | --- |
|  | **Vaccine Acceptance** | |  |
| **Survey question** | **Yes (n = 89)** | **No/ I don’t know (n = 140)** | **P-value** |
| Have you heard about the COVID-19 vaccine? |  |  | 0.49 |
| Yes | 82 (92.1%) | 129 (92.1%) |  |
| No | 7 (7.9%) | 9 (6.4%) |  |
| Don’t know | 0 | 2 (1.4%) |  |
| Do you believe the vaccine is effective in preventing COVID-19 infection? |  |  | <0.001* |
| Yes | 32 (36.0%) | 12 (8.6%) |  |
| No | 12 (13.5%) | 29 (20.7%) |  |
| Don’t know | 45 (50.1%) | 99 (70.7%) |  |
| Have you already taken the vaccine? |  |  | N/A |
| Yes | 14 (15.7%) | No (100%) |  |
| No | 75 (84.3%) | 0 |  |
| If you could take it right now, would you? (only asked to those who had not already been vaccinated, n = 215) |  |  | N/A |
| Yes | 75 (100%) | 0 |  |
| No | 0 | 94 (67.1%) |  |
| Don’t know | 0 | 46 (32.9%) |  |
| Who would you take the vaccine from? (n = 75) |  |  | N/A |
| Haitian health workers | 65 (86.7%) | 0 |  |
| Don’t know | 5 (6.7%) | 0 |  |
| International health workers | 1 (1.3%) | 0 |  |
| No preference | 4 (5.3%) | 0 |  |
| Would you recommend the vaccine to your family? |  |  | <0.001 |
| Yes | 71 (79.8%) | 18 (12.9%) |  |
| No | 1 (1.1%) | 84 (60.0%) |  |
| Don’t know | 3 (3.4%) | 32 (22.9%) |  |
| I’m not sure | 0 | 6 (4.3%) |  |
| Do you think the vaccine will prevent you from getting COVID-19? |  |  | <0.001 |
| Yes | 36 (40.5%) | 14 (10.0%) |  |
| No | 13 (4.6%) | 44 (31.4%) |  |
| Don’t know | 40 (44.9%) | 82 (58.6%) |  |
| Who do you think should get the COVID-19 vaccine? |  |  | <0.001 |
| Everyone | 78 (87.6%) | 74 (52.9%) |  |
| Don’t know | 9 (10.1%) | 56 (40.0%) |  |
| Only high risk people | 2 (2.3%) | 6 (4.3%) |  |
| No one | 0 | 3 (2.1%) |  |

| **Supplementary Table S7. Fear of COVID infection** | | | | | | |
| --- | --- | --- | --- | --- | --- | --- |
|  | **I am afraid I or my family will be infected with COVID-19** | | | | |  |
| **Survey question** | **strongly agree**  **(n = 20)** | **agree**  **(n = 84)** | **neutral**  **(n = 4)** | **disagree**  **(n = 85)** | **strongly disagree**  **(n = 53)** | **P-value** |
| Can COVID-19 be fatal? | | | | | | 0.008 |
| Yes | 1 (5.0%) | 25 (29.8%) | 1 (25.0%) | 23 (27.1%) | 13 (24.5%) |  |
| No | 16 (80.0%) | 44 (52.4%) | 1 (25.0%) | 41 (48.2%) | 38 (71.7%) |  |
| I don’t know | 3 (15%) | 15 (17.9%) | 2 (50.0%) | 21 (24.7%) | 2 (3.8%) |  |
| Would you recommend the vaccine to your family? | | | | | | 0.027 |
| Yes | 12 (63.2%) | 34 (49.3%) | 1 (25.0%) | 22 (30.6%) | 20 (39.2%) |  |
| No | 4 (21.0%) | 18 (26.1%) | 2 (50.0%) | 34 (47.2%) | 27 (52.9%) |  |
| I don’t know | 3 (15.8%) | 17 (24.6%) | 1 (25.0%) | 16 (22.2%) | 4 (7.8%) |  |
| Who do you think should get the vaccine? | | | | | | 0.027 |
| Everyone | 17 (85.0%) | 58 (69.1%) | 2 (50.0%) | 47 (56.0%) | 35 (66.0%) |  |
| Only people at risk of severe illness | 0 | 5 (6.0%) | 0 | 3 (3.6%) | 2 (3.8%) |  |
| No one | 2 (10.0%) | 0 | 0 | 1 (1.2%) | 0 |  |
| I don’t know | 1 (5.0%) | 21 (25.0%) | 2 (50.0%) | 33 (39.3%) | 16 (30.2% |  |
